# Kinetic and Parametric Analysis of the Separation of Ultra-Small, Aqueous Superparamagnetic Iron Oxide Nanoparticle Suspensions under Quadrupole Magnetic Fields

**DOI:** 10.3390/mi14112107

**Published:** 2023-11-17

**Authors:** Stefano Ciannella, Xian Wu, Cristina González-Fernández, Bahareh Rezaei, Jacob Strayer, Hyeon Choe, Kai Wu, Jeffrey Chalmers, Jenifer Gomez-Pastora

**Affiliations:** 1 Department of Chemical Engineering, Texas Tech University, Lubbock, TX 79409, USA; stefano.ciannella@ttu.edu (S.C.); cristina.gonzalezfdez@unican.es (C.G.-F.); 2 William G. Lowrie Department of Chemical and Biomolecular Engineering, The Ohio State University, 151 W. Woodruff Ave., Columbus, OH 43210, USA; wu.4073@buckeyemail.osu.edu (X.W.); strayer.117@buckeyemail.osu.edu (J.S.); choe.123@buckeyemail.osu.edu (H.C.); chalmers.1@osu.edu (J.C.); 3 Departamento de Ingenierías Química y Biomolecular, Universidad de Cantabria, Avda. Los Castros s/n, 39005 Santander, Spain; 4 Department of Electrical and Computer Engineering, Texas Tech University, Lubbock, TX 79409, USA; barezaei@ttu.edu (B.R.); kai.wu@ttu.edu (K.W.)

**Keywords:** superparamagnetic iron oxide nanoparticles (SPIONs), cooperative magnetophoresis, self-assembly, magnetic sedimentation, quadrupole magnetic sorter (QMS), kinetic modeling

## Abstract

Superparamagnetic iron oxide nanoparticles (SPIONs) have gathered tremendous scientific interest, especially in the biomedical field, for multiple applications, including bioseparation, drug delivery, etc. Nevertheless, their manipulation and separation with magnetic fields are challenging due to their small size. We recently reported the coupling of cooperative magnetophoresis and sedimentation using quadrupole magnets as a promising strategy to successfully promote SPION recovery from media. However, previous studies involved SPIONs dispersed in organic solvents (non-biocompatible) at high concentrations, which is detrimental to the process economy. In this work, we investigate, for the first time, the magnetic separation of 20 nm and 30 nm SPIONs dispersed in an aqueous medium at relatively low concentrations (as low as 0.5 g·L^−1^) using our custom, permanent magnet-based quadrupole magnetic sorter (QMS). By monitoring the SPION concentrations along the vessel within the QMS, we estimated the influence of several variables in the separation and analyzed the kinetics of the process. The results obtained can be used to shed light on the dynamics and interplay of variables that govern the fast separation of SPIONs using inexpensive permanent magnets.

## 1. Introduction

Magnetic nanoparticles (MNPs) denote a wide umbrella of materials that covers nanometer-sized particles below the threshold of 100 nm synthesized from magnetic materials like cobalt, nickel, iron, and their oxides [1,2,3]. Below certain dimensions, MNPs exhibit a magnetic trait named superparamagnetism [4], a quantum-originated effect associated with causing high magnetization values (hence the suffix ‘super’) when the particles are exposed to strong external magnetic fields. Their paramagnetic characteristic dictates that, upon removal from the vicinity of a magnetic source (null field condition), the MNP magnetization turns to zero since their single magnetic domain becomes incapable of aligning with an external field due to the rapid random flipping of its direction [5]. In other words, MNPs are not magnetized while off the influence of a magnetic field.

The magnetophoresis of MNPs refers to their motion in a viscous medium driven by an external magnetic field gradient [6,7,8], which is considered the relying principle of magnetic separation [9]. In particular, MNPs possess magnetic properties that allow them to be actively guided toward the direction of the external magnetic field gradient, resulting in an effective separation from the surrounding medium. Moreover, this technique offers the advantage of operating at ambient temperatures, further adding to its appeal and potential for various biological and engineering applications, especially in the fields of biomedicine, nanomedicine, bioengineering, and chemical engineering [10,11,12]. Nonetheless, it is important to highlight that a uniform magnetic field is not capable of causing the motion of magnetic nanoparticles, so a non-null magnetic field gradient ∇B (or ∇H in several references) is currently considered the main driver of magnetophoresis [13], which can be accelerated via the cooperative behavior of MNPs when certain physical criteria are met. Cooperative magnetophoresis relies on induced dipole–dipole interactions between MNPs [14,15,16] to form larger bodies, whether shaped as chains or clusters, a phenomenon also known as self-assembly, magnetic clustering effect, or field-induced reversible aggregation [17,18]. This effect is known for accelerating magnetophoresis and, consequently, speeding up the separation process of MNPs [19].

If cooperative magnetophoresis occurs, the magnetic nanoparticles initially aggregate under the influence of a magnetic field. Then, the particle clusters formed tend to migrate toward the direction of the magnetic field gradient. As the clusters migrate, they collide with other MNPs, resulting in the growth of larger aggregates [20]. These augmented aggregates exhibit increased magnetic forces and magnetophoretic velocity, attributed to their higher amount of magnetic material. This augmented velocity helps to counteract opposing forces, including viscous drag forces and random thermal fluctuation [21], resulting in observable outcomes within minutes or hours, depending on the particle size [17]. The theoretical premises involving the aggregation of magnetic particles can be extended to MNPs dispersed in an aqueous medium, where they can form particle clusters due to the influence of van der Waals and magnetic dipole–dipole interactions [22].

The separation of ultra-small (diameter < 50 nm) superparamagnetic iron oxide nanoparticles (SPIONs) generally requires expensive separators that make use of either complex columns and energy-costly electromagnets or inexpensive, permanent magnet-based devices that require hours or days to accomplish the separation [17]. High-gradient magnetic separation (HGMS) columns are particularly renowned for their ability to achieve high recovery rates. This is primarily accomplished via the magnetization of an internal metallic matrix via the utilization of electromagnets and superconducting magnets. These magnets can generate notably high magnetic gradients, thereby significantly augmenting the magnetic forces acting upon the particles [1]. Nevertheless, HGMS does exhibit certain limitations, especially concerning installation and operational expenses [23]. Furthermore, the presence of the matrix introduces complexities associated with the generation of inhomogeneous magnetic fields and forces within the column, resulting in challenges concerning the accurate description and realistic numerical simulation [24]. As a result, recent research endeavors have been directed toward investigating a cost-effective alternative to HGMS-based systems, known as low-gradient magnetic separation (LGMS). LGMS techniques have demonstrated the capability to achieve relatively swift separations by applying external field gradients typically lower than 100 T·m^−1^ [14,15,16,18]. Differently from this study, these LGMS investigations generally employ small permanent magnets, and separation occurs in hours or days. While these studies play a pivotal role in the development of permanent magnet-based magnetophoretic separators, further research is necessary to comprehensively elucidate the underlying mechanisms governing magnetic sorting processes under low to medium gradients and to optimize the working conditions to achieve a fast and complete separation using inexpensive permanent magnet systems.

**Figure 1:**
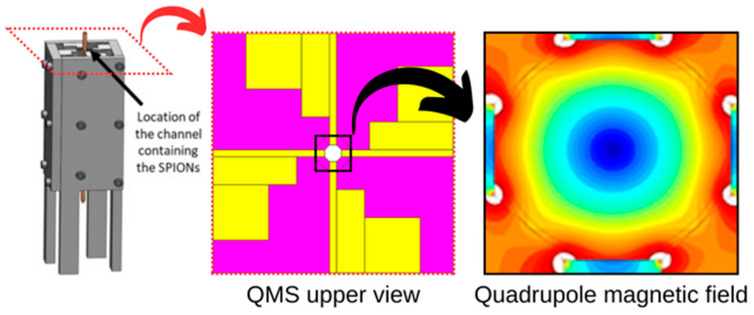
Depiction of our experimental setup and magnetic field pattern generated within the bore (channel) of the QMS (adapted from [17]).

Our previous works [17,25] revealed the potential self-assembly and magnetic separation of SPIONs with sizes below 50 nm employing quadrupole magnetic sorters (QMS), which consist of four permanent magnets placed in a quadrupole orientation that generates a high and constant field gradient. The magnetic field generated within the bore of the QMS is depicted in Figure 1, where at r=0, the field is null (center of the bore), and at r=R, the field is maximum (separator wall). We were able to separate ultra-small SPIONs with these inexpensive devices in a matter of minutes. However, these studies employed organic ferrofluids at high concentrations that (i) are noncompatible with biological applications, i.e., the suspensions used toxic organic solvents (chloroform/toluene), and (ii) use a high concentration of MNPs (up to 25 g∙L^−1^), which is not beneficial for the process economy.

The present study follows a similar approach as we explore the magnetic separation of 20 nm and 30 nm SPIONs dispersed in an aqueous medium at relatively low concentrations. To quantify the extent of the separation in the suspension, we evaluate the volume of the medium free of particles in the vessel via distinct intervals of exposition to a uniform magnetic field gradient ∇H. The kinetics of the separation process are also explored via the estimation of kinetic parameters, which are essential to properly model the evolution of concentration with time. Additionally, our previous analysis of the magnitude of the magnetophoresis [17,25] is enhanced by adding a second dimension in the estimation of concentration profiles, and therefore we present two-dimensional concentration maps for the entire vessel achieved via simple image acquisition and processing procedures.

This paper is structured as follows. After this introduction, Section 2 brings a summary of theoretical aspects and meaningful physical quantities related to magnetic separation. Next, Section 3 provides a thorough description of the methods employed in this study to (i) adequately prepare SPIONs aqueous solutions to undergo magnetic separation, (ii) properly operate the separation experiments via specific equipment, and (iii) estimate numerical values for variables of interest related to the experiments. In Section 4, we organize, present, and discuss the results and findings from the performed analyses. Finally, we finish with the main conclusions from the collected observations.

## 2. Theoretical Aspects of Magnetic Separation

### 2.1. Magnetic Quantities

The magnetic force $F_{\mathrm{mag}}$ (N) exerted by a magnetizing field H (A∙m^−1^) on a saturated particle with a diameter $d_{p}$ (m) is proportional to its volume $V_{p}$ (m^3^) as follows [18]:

(1) $$F_{\mathrm{mag}}=\mu_{0}V_{p}\left(M_{p}\cdot \nabla \right)H,$$

where $M_{p}$ is the saturation magnetization (A∙m^−1^), $\mu_{0}$ is the permeability of free space (4π × 10^−7^ H∙m^−1^), and ∇H is the magnetic field gradient (A∙m^−2^). The combined force acting on an agglomeration of nanoparticles is larger than the force acting on a single particle, meaning that the magnetic force expressed in Equation (1) will scale as clustering occurs by a factor of $N^{*}$ (aggregation parameter) [5], which is a function of the volumetric fraction and particle size [13]. Briefly, the cluster’s volume can be approximated as follows [21]:

(2) $$V_{c}=V_{p}N^{*}\left(\varphi_{0},d_{p}\right)=\frac{4}{3}\pi r_{p}^{3}{\cdot N}^{*}\left(\varphi_{0},d_{p}\right),$$

where $\varphi_{0}=c_{p}^{m}/\rho_{p}$ is the volume fraction of particles in the dispersion, $c_{p}^{m}$ is the mass concentration of particles (g∙L^−1^), $\rho_{p}$ is the particle density (kg∙m^−3^), $d_{p}$ (m) is the particle diameter, and $r_{p}$ (m) is the particle radius. The formal development of $N^{*}$ is rooted in a thermodynamic perspective that encompasses both the energetic and entropic aspects of particle dispersions. This approach takes into account key variables such as concentration and temperature [15]. The critical value of $N^{*}$ (for field-induced self-assembly) is $N^{*}=1$; when $N^{*}<1$, the formation of structures is not feasible as entropy dominates. On the other hand, for $N^{*}>1$, the particles will likely arrange themselves into various chains and clusters, depending on the magnitude of $N^{*}$ [5]. Finally, the aggregation parameter can be computed as follows:

(3) $$N^{*}={\left[\varphi_{0}\exp\left(\Psi -1\right)\right]}^{1/2}$$

The term Ψ in Equation (3) is the magnetic coupling parameter (also represented by Γ), which represents a ratio between the magnetic dipole–dipole interaction energy and thermal energy [25]:

(4) $$\Psi =\frac{\mu_{0}\pi r_{p}^{3}{M_{p}}^{2}}{9k_{B}T},$$

where $k_{B}$ is the Boltzmann constant (1.3806 × 10^−23^ J∙K^−1^) and T (K) is the absolute temperature. This ratio relates to the occurrence of cooperative magnetophoresis and must be greater than one. Concerning the magnetic separation of SPIONs, the size of the magnetic particle holds considerable significance. Given the infinitesimally small size of SPIONs, hampering effects on the magnetophoresis in the form of viscous drag and the entropy-driven random thermal energy can take place depending on the particle size [2,17], with the latter based on Brownian motion. Challenging separations can take form when the Fe_3_O_4_ MNP size falls below a specific threshold of around 40 nm [26], as the randomizing thermal energy will predominate over magnetic energies, thus hindering the magnetically directed movement of the particles. For this reason, particle aggregation or self-assembly is critical for magnetic separation to occur in a fast manner. Specific length scales can be incorporated to determine the type of self-assembly kinetics, which can be either dominated by magnetic interactions or random diffusion. The rationale is straightforward and involves a comparison of the average distance between particles $d_{p\text{-}p}$ prior to the application of a magnetic field [15,25]

(5) $$d_{p\text{-}p}=2.7\frac{d_{p}}{6\varphi_{0}^{1/3}},$$

and the distance $\lambda_{B}$ related to a balanced relation between the magnetic attractive and thermal energies of two aligned dipoles (i.e., Ψ=1), known as the magnetic Bjerrun length [25]:

(6) $$\lambda_{B}=d_{p}{\cdot \Psi }^{1/3}$$

The relation between $d_{p\text{-}p}$ and $\lambda_{B}$ will determine the type of aggregation kinetics of the SPIONs [13], meaning that for $\lambda_{B}\gg d_{p\text{-}p}$, or $\varphi_{0}\Psi \gg 0.1$, magnetic interactions dominate and particle self-assembly is facilitated. In contrast, for $\lambda_{B}\ll d_{p-p}$, or $\varphi_{0}\Psi \ll 0.1$, diffusive mechanisms dominate and are characterized by magnetic interactions happening within random encounters of particles in their diffusive paths governed by Brownian motion; in other words, cooperative magnetophoresis is not facilitated. Notably, the magnetic separation of SPIONs points toward demanding self-assembly kinetics dominated by dipole–dipole magnetic interactions.

### 2.2. Separation Kinetics

The kinetic laws considered in this study are a time-dependent relationship that describes the evolution of estimated SPION concentrations toward a new equilibrium state caused by the exposure to the magnetic gradient ∇H, therefore assuming the form of an asymptotic decreasing exponential curve. These dynamic models are widely used in a diverse range of separation processes [27,28,29] and, among them, we highlight the first-order and second-order models given by Equations (7) and (8), respectively:

(7) $$\dot{c}\left(t\right)=k_{1}\cdot c\left(t\right)$$

(8) $$\dot{c}\left(t\right)=k_{2}\cdot {c\left(t\right)}^{2}$$

where $c\left(t\right)$ (g∙L^−1^) represents the time-dependent concentration term, $\dot{c}\left(t\right)$ (g∙L^−1^∙min^−1^) denotes the temporal rate of concentration decline, and $k_{1}$ (min^−1^) and $k_{2}$ (L∙min^−1^∙g^−1^) are the kinetic constants. Finally, their linearized algebraic solutions for the initial-value problem with $c\left(t=0\right)=c_{0}$ and $c\left(t>0\right)=c\left(t\right)$ are

(9) $$\ln\left[\frac{c\left(t\right)}{c_{0}}\right]=-k_{1}t$$

and

(10) $$\frac{1}{c\left(t\right)}-\frac{1}{c_{0}}=k_{2}t$$

## 3. Materials and Methods

### 3.1. Sample Preparation

The SPIONs (catalog numbers SPA20 and SPA30) used in this study were acquired from Ocean Nanotech (San Diego, CA, USA) and have a uniform diameter of 20 nm and 30 nm, respectively. The particles are covered by a single polymeric coat, according to the manufacturer. The magnetite (Fe_3_O_4_) SPIONs were acquired in the form of biocompatible (aqueous) dispersions whose initial concentration is 5 g∙L^−1^, in contrast with previous studies that employed SPIONs suspended in an organic non-biocompatible solvent (e.g., chloroform/toluene) at higher initial concentration values of 25 g∙L^−1^ [17,25]. It is assumed that the particle density is approximately 5000 kg∙m^−3^ [17]. To reach the experimented concentration values of 0.5 and 2.0 g∙L^−1^, the stock solution was diluted in purified water from a Milli-Q^®^ Direct 8/16 System (Millipore Sigma, Burlington, MA, USA). The stock concentration of 5 g∙L^−1^ was also experimented with, yet no dilution was required. To serve as the reservoir for the separation of SPIONs within the QMS bore, we utilized square glass channels (1 mm Ø, 1.4 mm Ø; VitroCom, catalog number 8100–600) 60 mm in height and bottom melted before the injection of the SPION solution. These square tubes were then filled up to approximately half their length (30 mm) with the diluted solutions of SPIONs using a 20G polypropylene precision tip plugged into a 1 mL plastic syringe. Finally, the prepared sample was sealed at the top with Parafilm^®^ M (Millipore Sigma, Burlington, MA, USA) to prevent inaccuracies in mass concentration measurements by evaporation of the water solvent.

### 3.2. Operation of Magnetic Separation Experiments

**Figure 2:**
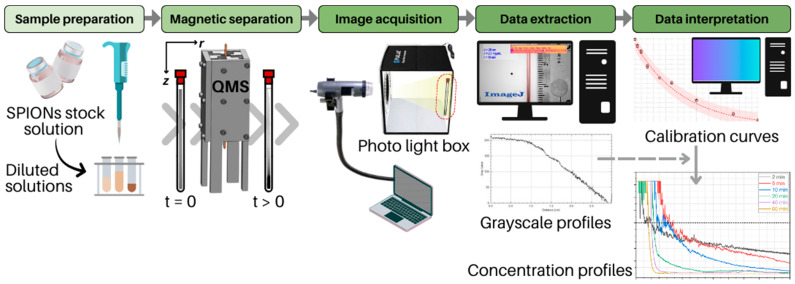
Operation of magnetic separation experiments: from sample preparation to estimation of SPION concentration profiles.

The magnetic separation, sample image acquisition, and grayscale measurement methodologies employed in this study were conducted following previously published investigations that utilized a QMS [17,25]. Briefly, the prepared samples (glass channel filled with SPIONs solution of known concentration) were introduced into the bore of the quadrupole arrangement, where the magnetic field strength B on the QMS inner wall and constant radial gradient B/r, or ∇B, are 1.36 T and 286 T∙m^−1^, respectively. Inside the QMS, the SPIONs are subjected to magnetophoresis in the radial direction (direction of the magnetic field gradient), particle self-assembly, and sedimentation due to gravity. Therefore, increased particle concentrations are expected at the bottom of the tubes with time as clusters are being formed. Six distinct exposure times (2, 5, 10, 20, 40, and 60 min) were allotted for the samples to remain inside the QMS, after which they were completely removed upon completion of the magnetic exposure times. The experiments were conducted isothermally at room temperature of approximately 20 °C. Next, the sample was positioned vertically on the inner back wall of a photo light box with a white background to undergo image acquisition. A portable digital microscope (Dino-Lite Edge 140x 5MP, Dunwell Tech Inc., Torrance, CA, USA; model AM73515MZTL) was used for image capturing and was positioned at the photo light box opening where a white LED light circle strip of adjustable intensity provided the proper illumination. Finally, three monochromatic pictures were taken for every experimented condition with the aid of software provided by the microscope manufacturer (DinoLite Capture 2.0, https://www.dinolite.us/download/, accessed on 17 January 2023). The experimental conditions are available in Table S1 in the Supplemental Information. The acquired images were then converted to an 8-bit format and processed to collect meaningful quantitative measurements that directly correlate to concentration profiles generated by the magnetophoresis of SPIONs. In this case, grayscale pixel values were consistently measured using ImageJ freeware v1.54g (https://imagej.nih.gov/ij/, accessed on 1 December 2022). To estimate the SPIONs concentration profile for each run, three grayscale measurements (one observation with two replicates) were performed along the walls and center of the tube in the axial direction, separately. To assist in the estimation of concentration values in the sample, a calibration curve for each SPION size used in this study was constructed before the conduction of magnetic separation. Their design consisted of associating average grayscale values for a series of known fixed SPION concentrations and finally fitting a nonlinear model that was later used in the magnetophoresis experiments to obtain concentration values from grayscale measurements (see Tables S2 and S3 and Figures S1 and S2 in the Supplementary Materials). A summary of the described magnetic separation experiments is depicted in Figure 2, while Figure S3 (Supplementary Materials) shows the assembled magnetic separation experimental setup with a QMS.

### 3.3. Estimation of Variables of Interest

**Figure 3:**
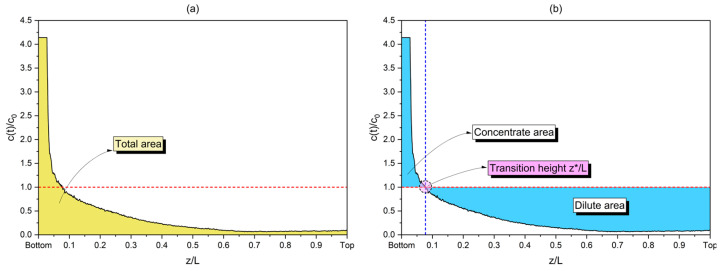
Estimated areas from axial concentration profiles: (**a**) the total area under the concentration profile; (**b**) concentrated and dilute areas separated by the dimensionless transition height $z^{*}/L$, where L represents the filled length of the sample (30 mm).

This study employed a method to estimate numerical values of SPION mass concentrations in aqueous media based on quantities derived from image analysis procedures. Particularly, individual grayscale pixel values were used as input to perform this estimation via calibration curves that admitted a continuous range of grayscale pixel intensities from 1 to 255 arbitrary units, which corresponds to an 8-bit image format. From the estimated concentrations, the dimensionless quantity $c(t)/c_{0}$ is hereby used to track the time development of magnetic separations over the tube length (z), where c(t) and $c_{0}$ are the estimated concentration at time t (which will differ depending on the position z) and the initial concentration (constant for the entire length or position z), respectively. By using a dimensionless ratio of estimated concentrations and their initial value at time zero, two parameters of interest are then distinguished in the separation profile: the concentrated ($c(t)/c_{0}>1$) and dilute ($c(t)/c_{0}<1$) regions. Figure 3 illustrates these regions.

By using this dimensionless concentration ratio, it becomes possible to denote numerical values for the dilute area of each separation experiment, which tends to increase as the magnetic exposure time tends to an extensive period and therefore is an intuitive measurement of the separation development. The point $z^{*}/L$ in the nondimensional axis z/L can be approximated as to the dimensionless height where the system transitions from the concentrated to the dilute region for each condition, and therefore can also be estimated via this method.

### 3.4. Estimation of Kinetic Constants

The lowest concentration within the dilute region was used as the working variable c(t) on the left-hand side of Equations (9) and (10), which can be found within the proximity of the top portion of the glass channel, where grayscale values are close to their maximum after separation has occurred. We assumed that the dynamics in the dilute region evolve toward equilibrium as the magnetic exposure time increases. This assumption allowed us to perform linear data fitting procedures for both first-order and second-order kinetic models using the dilute region as a reference for concentration values. Several statistical quantities were evaluated for each combination of particle size and initial concentration, namely the statistical significance of the slope, the adjusted *R*^2^ parameter, the normality of model residuals, and the magnitude of Shapiro–Wilk’s W, which infers on the probability of randomly observing a normal distribution of model residuals from a sample of a certain size [30]. Residuals that follow a normal distribution are due to pure random error and therefore should be essentially random.

## 4. Results and Discussion

To gain insights into the mechanisms governing SPIONs separation using a horizontal (perpendicular to gravity) magnetic gradient, particularly at relatively low concentrations and using biocompatible, viscous, and aqueous solutions, a diverse range of conditions was examined. To organize both the conducted investigations and their respective findings, the next subsections address the following aspects: (i) the depiction of SPION estimated concentration profiles as a function of magnetic exposure time, (ii) the determination of kinetic parameters of the separation of particles from the surrounding aqueous media, and (iii) the effects of experimented variables via analysis of variance (ANOVA) procedures.

### 4.1. Estimated SPIONs Concentration Profiles

**Figure 4:**
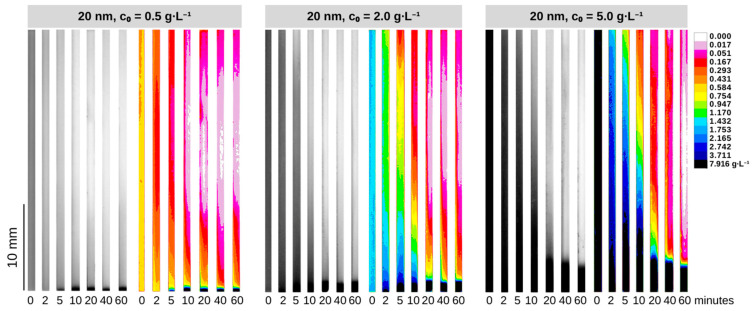
Raw 8-bit pictures of magnetically separated suspensions and their respective two-dimensional maps for the 20 nm SPIONs at different values of $c_{0}$.

**Figure 5:**
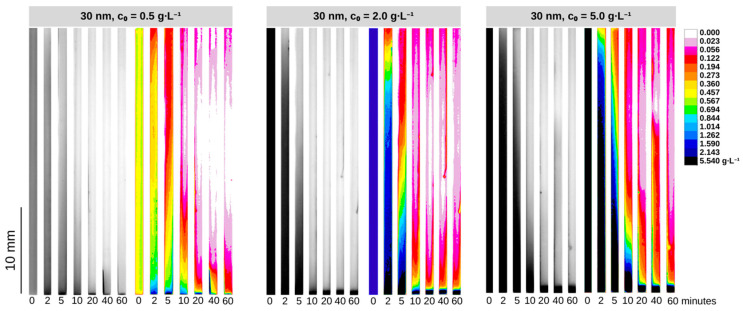
Raw 8-bit pictures of magnetically separated suspensions and their respective two-dimensional maps for the 30 nm SPIONs at different values of $c_{0}$.

**Figure 6:**
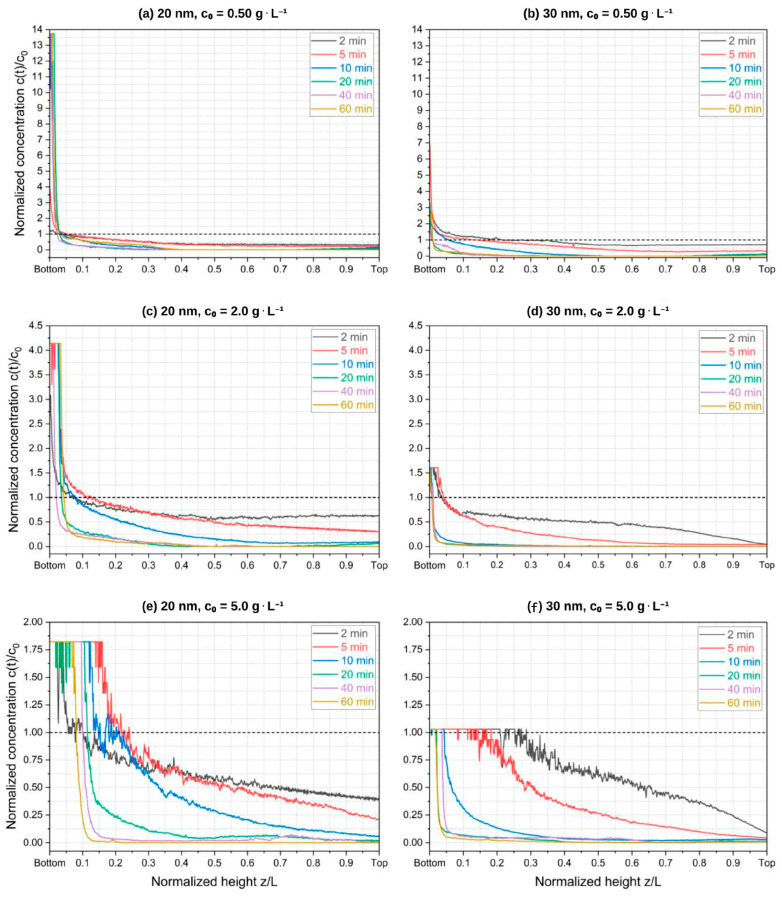
Estimated concentration profiles of 20 nm and 30 nm SPIONs in aqueous media: experimented fixed parameters are B=1.36 T at the QMS inner wall, ∇B=286 T∙m^−1^, T=20 °C, and L=30 mm. The observed plateaus for high concentration values ($c\left(t\right)>$ 5.0 g∙L^−1^) are due to the limited measurement capability of our method using grayscale pixel intensity values.

To corroborate the superior efficiency of our QMS in comparison to a regular permanent magnet, we included an additional test (Figure S4 of Supplementary Materials) performed using a single NdFeB N42 permanent magnet to carry out the magnetic separation of 20 and 30 nm SPIONs. The QMS showed superior performance in separating the nanoparticles via an aggregation–sedimentation mechanism (Figure S5 of Supplementary Materials). Figure 4 and Figure 5 show the concentration dynamics of SPIONs during the magnetic separation process in the QMS in the form of 2D maps of estimated concentrations. ImageJ freeware was used to treat collected pictures of samples and convert them into concentration heatmaps using previously established calibration curves. Notably, the raw monochromatic 8-bit format pictures of samples taken after specific periods of magnetic exposure become meaningful via the application of a 16-color spectrum filter based on greyscale pixel intensity levels, allowing a diverse range of different concentrations to be distinguishable and visible. Figure 6 brings the time-wide estimated concentration profiles of 20 nm and 30 nm SPIONs for different values of $c_{0}$. These profiles were estimated at the center of the glass channel, which, along with the surface of the wall toward where the nanoparticles migrated due to magnetophoresis (the direction of the magnetic field gradient), are the regions of interest that have been addressed in our previous studies using SPIONs.

One can notice a maximum $c/c_{0}$ ratio near the channel’s bottom for all the conditions tested, which is in agreement with the 2D maps presented in Figure 4 and Figure 5. Notably, the experiments involving the lowest initial concentration ($c_{0}=0.50$ g∙L^−1^) showed that higher $c(t)/c_{0}$ values at the bottom are achieved after a few minutes of magnetic exposition with increasingly higher grayscale values around the top region of the channel (i.e., becoming clearer), indicating progressive dilution and a consequent expansion of the dilute area. This is due to inaccuracies in determining concentrations above 5 g∙L^−1^ with our calibration curves. It is also noticeable that the highest initial concentration experiments for the 30 nm particles promoted a faster evolution toward achieving equilibrium in the *z* coordinate. As $c_{0}$ is increased, the concentration profiles do not change considerably over *z* after 10 min of exposition to the magnetic field, thus indicating faster kinetics toward the largest initial concentration. It might be possible that the concurrent occurrences of particle self-assembly and gravitational sedimentation are associated with the kinetics dependence of $c_{0}$ as demonstrated by the separation data in Figure 6.

From Figure 6, one may infer that the variable $c_{0}$ has a certain effect on the temporal evolution of the estimated dilute area. One must also bear in mind that the larger particles are heavily likely to undergo cluster formation when exposed to the experimented magnetic fields. Table 1 presents the magnetic parameters presented in Section 2 (forces acting on the particles as well as other parameters related to the magnetic dipole–dipole and thermal energies) for our experimental conditions, and the effect of the particle size becomes evident. Cluster formation promotes a larger magnetophoretic force to exert motion on the nanoparticles toward the region of the maximum magnetic field, after which sedimentation can also occur in the direction of gravity. Thus, it is expected that larger SPIONs in more concentrated dispersions present faster kinetics. This theoretical premise is noticeable within the comparison of the plots of Figure 6: saturation or equilibrium (in the form of $c(t)/c_{0}$ plateaus) is achieved quicker for the 30 nm SPIONs. Saturation, in this context, means the maximum measured value of $c(t)/c_{0}$ that can be estimated at the bottom of the channel ($z<z^{*}/L$), as well as the minimum $c(t)/c_{0}$ that is measured at the top of the channel ($z>z^{*}/L$), via grayscale pixel intensity values. In other words, it means how quickly the concentrated and dilute areas started to show fixed normalized concentration values.

One can see from Table 1 that the volume fraction $\varphi_{0}$ significantly impacts the distance $d_{p\text{-}p}$, resulting in a progressively smaller void between particles as the initial concentration rises. This effect enhances the dipole–dipole interaction energy, facilitating quicker separation kinetics by promoting particle self-assembly as $N^{*}$ increases. Combining this physical premise with the predefined concept of observed saturation, it can be inferred from the data presented in Figure 6 that the separation of 30 nm SPIONs demonstrated potentially faster kinetics compared to their 20 nm counterparts. Specifically, in the case of the 30 nm SPION solution of $c_{0}$ = 5 g∙L^−1^, saturation can occur within 10 min of exposure time, while in the case of 20 nm at the same initial concentration, saturation was observed within 20 min of magnetic exposure. Interestingly, this small difference in the saturation time contrasts with the considerable (several orders of magnitude) discrepancy in the values of $N^{*}$ displayed in Table 1. As the exposure time extends, the dilute areas become slightly more evident for both 20 nm and 30 nm particles, further supporting the observation of a modest positive relationship between kinetic parameters and SPIONs size.

**Figure 7:**
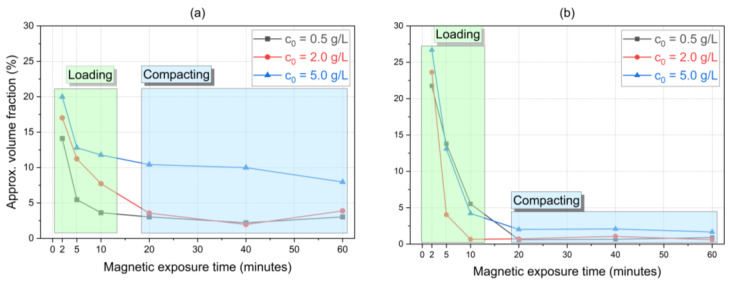
Dynamics of estimated volume fraction of the concentrated area for (**a**) 20 nm and (**b**) 30 nm SPIONs in water: during the loading phase, SPIONs migrate to the channel’s bottom where sedimentation takes place and will then assume a following compacting mechanism, here represented by an approximately constant volume fraction for extended magnetic exposure times.

Furthermore, the approximate normalized transition heights $z^{*}/L$ observed in this study were converted to the approximate volume fraction occupied by the concentrated area and then organized in Figure 7. These curves represent the dynamic behavior of the vertical extension of the concentrated area. Based on these approximated values, we proposed a distinction between two sequential phenomena that described SPION magnetic sedimentation at the channel’s bottom for our tested conditions: loading and compacting. According to our data, the initial 20 min show a loading behavior at low z values (the particles migrate toward the gravity direction and start accumulating in the regions close to the channel bottom). Following this, extended magnetic exposure leads to the stabilization of the concentrated volume fraction around 20 min for both 20 nm and 30 nm SPION solutions at lower z values. This represents the compacting behavior where the concentration of the particles reaches the maximum at the bottom of the channel.

### 4.2. Separation Kinetics

**Figure 8:**
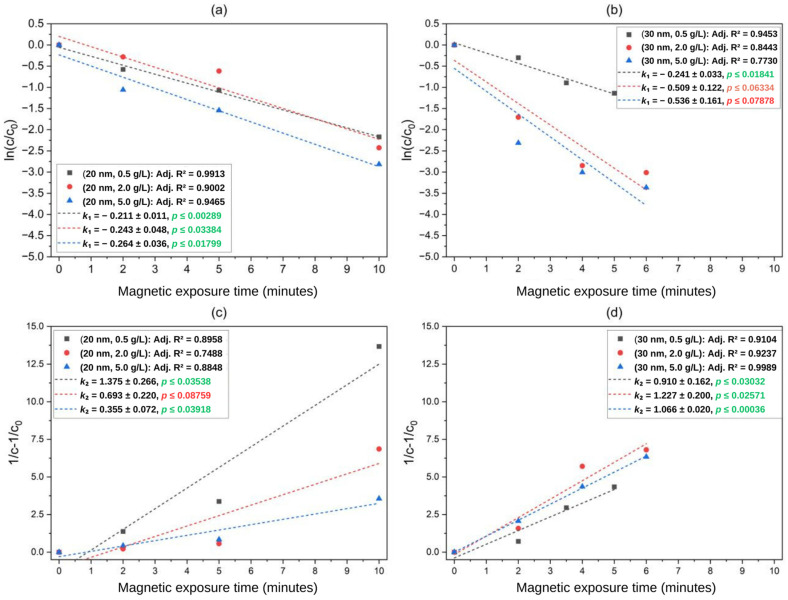
Linear fitting of the lowest concentration within the dilute region (c(t)) into first-order (first row) and second-order (second row) models: separation of (**a**,**c**) 20 nm and (**b**,**d**) 30 nm SPIONs. Kinetic constants whose *p*-values are highlighted in green are statistically significant, while the ones highlighted in red are statistically non-significant according to collected evidence.

The magnetic separation of MNPs under medium to low gradients has been explored in terms of kinetic modeling but with different types of magnet systems and arrangements [14,18,31]. To address the kinetics involved in the magnetic separation of SPIONs using our permanent magnet-based QMS, we calculated the normalized concentration $c(t)/c_{0}$ and the quantity $1/c\left(t\right)-1/c_{0}$ and used those for calculating the kinetic constants, as expressed in Equations (9) and (10). In these models, the kinetic constant k dictates the pace of the magnetic separation: for the first-order model, the reciprocal of $k_{1}$ is purely a time constant, while for the second-order model, the constant $k_{2}$ has units of g^−1^∙L∙min^−1^. Table 2 and Figure 8 collectively elucidate the main results of the fitting procedures.

Based on the observations described in Section 4.1, we hypothesized that the time required for separation has a certain dependence on the particle size and the initial concentration of particles dispersed in the aqueous solutions. Particularly, changes in initial concentration directly affect the interparticle distance $d_{p\text{-}p}$, the magnetic Bjerrun length, and the volume fraction, along with the aggregation parameter $N^{*}$. The kinetic constants might be associated with changes in $c_{0};$ therefore, we searched for physical evidence to support the hypothesis of this dependence. The confidence level α was constant and equal to 0.05 for statistical significance. According to the results in Table 2, the magnetic separation of 20 nm SPIONs was overall slightly accelerated by increasing the initial concentration $c_{0}$, regardless of the type of model. By taking the reciprocal of $k_{1}$, one can notice that the resulting time constant assumes its highest and lowest values when $c_{0}=0.5$ and $c_{0}=5.0$ g∙L^−1^, respectively, indicating a modest but observable increase in the kinetic constant and, therefore, in the rate of accumulation of particles at the bottom of the channel. For the larger particles, similar behavior is observed regarding the fitting into the first-order model, yet the first-order model results showed no apparent relation to changes in $c_{0}$. In this category, the second-order model showed an overall higher likelihood to better represent the time dependence of the magnetic separation. The fittings that achieved the best results in terms of adjusted *R*^2^ were the combinations (20 nm, 0.5 g∙L^−1^) and (30 nm, 5.0 g∙L^−1^), with *R*^2^ values of 0.991 and 0.999, respectively. One must also consider the slope significance and normality test metrics to decide upon a potential best fit: the *p*-value for the slope should be as low as possible, while Shapiro–Wilk’s W result should assume high values. In other words, there is evidence that magnetic separation kinetics is positively favored by the initial concentration in the case of 20 nm and 30 nm SPIONs in water. Next, the ANOVA results will determine the size of the effect (i.e., level of dependence) of the experimented variables on the estimated dilute area, whose size can be intuitively related to the level of progression of the magnetic sedimentation.

### 4.3. Parametric Analysis

**Figure 9:**
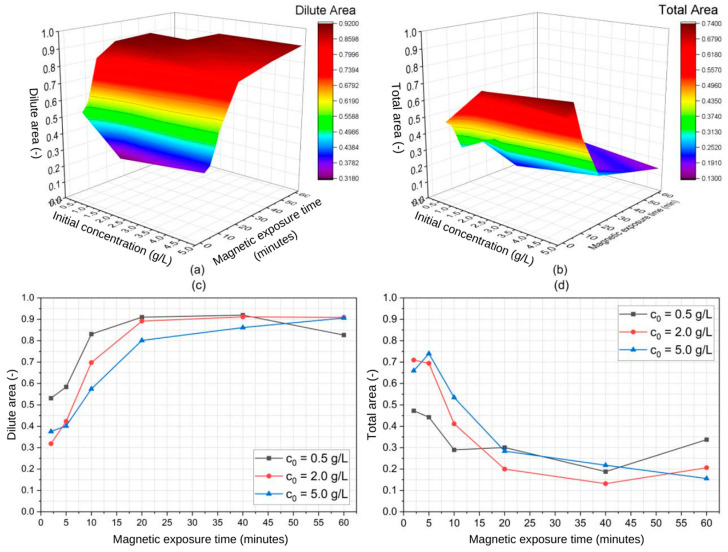
Three-dimensional plots of experimented variables for the 20 nm SPIONs regarding (**a**) estimated dilute area and (**b**) estimated total area along with concentration-wide respective 2D plots (**c**,**d**).

**Figure 10:**
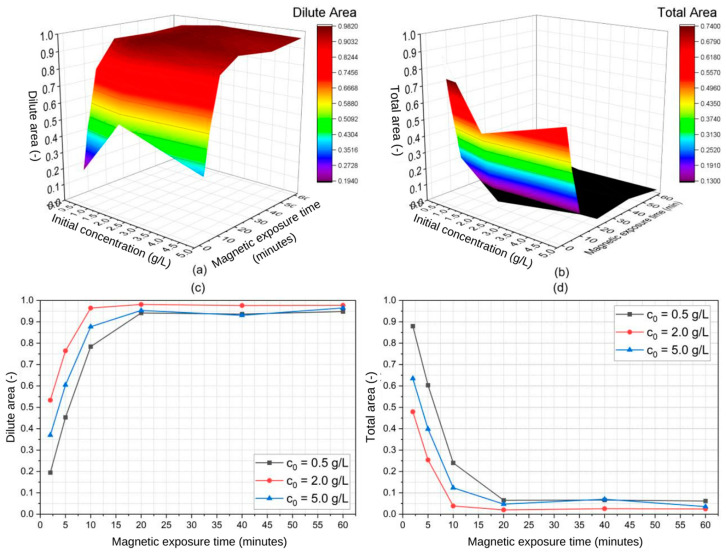
Three-dimensional plots of experimented variables for the 30 nm SPIONs regarding (**a**) estimated dilute area and (**b**) estimated total area along with concentration-wide respective 2D plots (**c**,**d**).

The parametric analysis of the magnetic separation of SPIONs in water was performed via ANOVA, which identified the statistically significant variables (i.e., p≤α) along with an estimate for the magnitude and direction of their effects (directly or inversely proportional). First, we plotted the estimated dilute and total areas as functions of the particle’s initial concentration and magnetic exposure time and performed ANOVA. These plots are depicted in Figure 9 and Figure 10 for the 20 nm and 30 nm SPIONs, respectively. Then, we used JMP Pro 16 software to quantify the coefficients of each variable (i.e., their effects) while keeping a level of significance constant at α = 0.05.

Table 3 shows the estimated effects of particle size, SPION initial concentration, and magnetic exposure time on the dilute and total areas presented in Figure 9 and Figure 10. Notably, the shapes of the curves in Figure 7 (concentrated area) are similar yet reciprocal to the ones presented in Figure 9 and Figure 10 (diluted area), given that the relationship between these concentrated and diluted areas is governed by the mass conservation principle. One can notice that the diluted area in Figure 10 (30 nm particles) reaches higher values and in a faster manner than the diluted area presented in Figure 9 (20 nm particles), evidencing the effect of the particle size in the separation. The opposite is observed with the total area.

From Table 3, it becomes evident that both particle size and magnetic exposure time have been identified as significant variables with positive effects. The experimented variables were arranged in a factorial manner such that three quadratic and one cubic term were addedin Table 3. Notably, particle size exerts an influence roughly five times smaller than that of exposure time. Nevertheless, it is worth mentioning that the estimated *p*-value for particle size is just on the border of statistical significance. Given that prior research has already indicated the profound effect that Fe_3_O_4_ nanoparticle size has in the context of magnetic separation [25,32], it is reasonable to conclude that our findings also suggest a size-dependent relation in the separation process using a QMS. Furthermore, despite the numerical differences that can be found in Table 2 regarding the estimated values of $k_{1}$ and $k_{2}$ for different initial concentrations, ANOVA results pointed at $c_{0}$ being a non-significant variable, meaning that not enough evidence could be collected to statistically differ the effect of this variable from a null one. Still, considering that the experiments were performed using a constant and equal sample volume across all runs, the magnetic exposure time is significant and therefore can be used to predict the extent of the separation process.

## 5. Conclusions

This work presented a comprehensive discussion of findings regarding the magnetic separation of commercial 20 nm and 30 nm Fe_3_O_4_ SPIONs dispersed in a biocompatible solution (water) at relatively low concentration values (0.5, 2, and 5 g·L^−1^) when exposed to a horizontal and homogenous field gradient generated by a QMS device. In this study, we introduced kinetic modeling to characterize the batch magnetic separation of ultra-small SPIONs within the QMS, and we were able to successfully collect kinetic modeling results from several experimented conditions by considering simple kinetic equations. In this sense, we provided insights into the estimation of separation kinetic parameters, specifically those of the time-dependent motion and particle accumulation on a collection plane. In practice, we demonstrated that the magnetic separation kinetic constant was slightly favored upon increasing the initial concentration of particles, indicating a modest positive dependency on the initial concentration for 20 nm and 30 nm SPIONs. We also observed a faster separation of the 30 nm (larger) particles. Furthermore, we statistically evaluated the significance of the estimated kinetic constants and assessed other statistical metrics, such as the normality of residuals and respective normality tests. A parametric analysis using ANOVA results was included to elucidate the statistical significance of the experimented variables and their effect on the volume fraction of the particle-free medium (or dilute region of the samples). These results collectively shed light on the intricate dynamics and interplay of variables governing the behavior of SPIONs within the system. We expect that the methodological approach of this study will be useful to further explore the separation mechanisms of MNPs and their improvement for practical applications.

## Figures and Tables

**Table 1:** Magnetic parameters of experimented particle sizes and initial concentrations of SPIONs.

$d_{p}$ (nm)	$c_{0}$ (g∙L^−1^)	$\varphi_{0}$ (-)	$F_{\mathrm{mag}}$ (fN)	$d_{p-p}$ (nm)	$\lambda_{B}$ (nm)	Ψ (-)	$N^{*}$ (-)
20	0.5	$1.0\times 10^{-4}$	$3.594\times 10^{-1}$	193.9	42.73	9.75	0.796
20	2.0	$4.0\times 10^{-4}$	$3.594\times 10^{-1}$	122.1	42.73	9.75	1.592
20	5.0	$10.0\times 10^{-4}$	$3.594\times 10^{-1}$	90.0	42.73	9.75	2.517
30	0.5	$1.0\times 10^{-4}$	1.213	290.8	96.15	32.92	$85.4\times 10^{3}$
30	2.0	$4.0\times 10^{-4}$	1.213	183.2	96.15	32.92	$170.8\times 10^{3}$
30	5.0	$10.0\times 10^{-4}$	1.213	135.0	96.15	32.92	$269.9\times 10^{3}$

**Table 2:** Estimated kinetic constants with respective statistical metrics.

$d_{p}$ (nm)	$c_{0}$ (g∙L^−1^)	Kinetic Constant	Adj. *R*^2^	Slope Significance	Norm of Residuals	Shapiro–Wilk Normality Test $\left(p\leq W\right)$
20	0.5	$k_{1}=-0.211\pm 0.011$	0.9913	p≤0.0029	0.1212	p≤0.3526
		$k_{2}=1.375\pm 0.266$	0.8958	p≤0.0354	2.8303	p≤0.2515
	2.0	$k_{1}=-0.243\pm 0.048$	0.9002	p≤0.0338	0.4885	p≤0.1720
		$k_{2}=0.693\pm 0.220$	0.7488	p≤0.0876	2.3449	p≤0.2701
	5.0	$k_{1}=-0.264\pm 0.036$	0.9465	p≤0.0179	0.3821	p≤0.7282
		$k_{2}=0.355\pm 0.073$	0.8848	p≤0.0392	0.7711	p≤0.1520
30	0.5	$k_{1}=-0.241\pm 0.033$	0.9453	p≤0.0184	0.1737	p≤0.1163
		$k_{2}=0.910\pm 0.162$	0.9104	p≤0.0303	0.8488	p≤0.0652
	2.0	$k_{1}=-0.509\pm 0.122$	0.8443	p≤0.0533	0.7748	p≤0.4665
		$k_{2}=1.227\pm 0.200$	0.9237	p≤0.0257	1.2690	p≤0.0456
	5.0	$k_{1}=-0.536\pm 0.161$	0.7730	p≤0.0788	1.0188	p≤0.3851
		$k_{2}=1.066\pm 0.020$	0.9989	p≤0.0004	0.1275	p≤0.5475

**Table 3:** Full factorial ANOVA results for collected magnetic sedimentation data.

	Output	Effect Estimates		*p*-Value	
Input		Diluted Area	Total Area	Diluted Area	Total Area
Particle Size		0.0374 ± 0.020	−0.0869 ± 0.021	0.0580	0.0001
Concentration		−0.0178 ± 0.020	−0.0002 ± 0.025	0.4600	0.9131
Exposure time		0.2302 ± 0.026	−0.2204 ± 0.027	<0.0001	<0.0001
Particle size · Concentration		0.031 ± 0.025	−0.0215 ± 0.025	0.1997	0.4043
Particle Size · Exposure time		−0.0092 ± 0.026	−0.0067 ± 0.027	0.7277	0.8106
Concentration · Exposure time		0.0006 ± 0.032	−0.0265 ± 0.033	0.8573	0.4277
Particle size · Concentration · Exposure time		−0.0345 ± 0.031	0.064 ± 0.033	0.2738	0.0658

The (·) character is used here to refer to multiplication.

## Data Availability

The data presented in this study are available upon request from the corresponding author.

## Supplementary Materials

The following supporting information can be downloaded at https://www.mdpi.com/article/10.3390/mi14112107/s1. Table S1: Experimental design; Table S2: Experimental data for 20 nm SPIONs calibration; Figure S1: 20 nm SPIONs calibration curve; Table S3: Experimental data for 30 nm SPIONs calibration; Figure S2: 30 nm SPIONs calibration curve; Figure S3: Experimental setup for magnetic separation of SPIONs using a QMS: (a) QMS and photo box, (b) frontal view of the QMS with a sample inserted in its bore, and (c) complete setup with image acquiring unit; Figure S4: The assembled magnetic separation control setup; Figure S5: Magnetic separation of (a) 20 nm SPIONs and (b) 30 nm SPIONs at an initial concentration of 1.0 g·L^−1^ for 60 min using the control setup and the QMS.
