# Supplementary material for: Kinetic and Parametric Analysis of the Separation of Ultra-Small, Aqueous Superparamagnetic Iron Oxide Nanoparticle Suspensions under Quadrupole Magnetic Fields
Source: Micromachines (Basel). 2023 Nov 17;14(11):2107. doi: 10.3390/mi14112107 (PMC10672830; doi:10.3390/mi14112107)
Supplement: Supplementary file 1 [file micromachines-14-02107-s001.zip › micromachines-2676396-supplementary.pdf]

micromachines

MDPI

Supplemental Material

Table S1. Experimental design

Particle size (nm)	SPION concentration ($g.L^{-1}$)	Exposure time (min)	Run
20	0.5	2	1
		5	2
		10	3
		20	4
		40	5
		60	6
	2.0	2	7
		5	8
		10	9
		20	10
		40	11
		60	12
	5.0	2	13
		5	14
		10	15
		20	16
		40	17
		60	18
30	0.5	2	19
		5	20
		10	21
		20	22
		40	23
		60	24
	2.0	2	25
		5	26
		10	27
		20	28
		40	29
		60	30
	5.0	2	31
		5	32
		10	33
		20	34
		40	35
		60	36

1 Calibration of 20 nm SPIONs

This section presents the mass concentration values of 20 nm SPIONs in aqueous solutions, along with their corresponding grayscale values. The measurements were conducted in triplicates and the arithmetic average was calculated for the entire length of the glass channel. Therefore, each row of the second columns of Table S2 contains the arithmetic average of grayscale intensity values for the entire length of the center of the channel.

Table S2. Experimental data for 20 nm SPIONs calibration

Mass concentration (g·L^{-1})	$\overline{G}$	$\overline{\overline{G}} \pm s$
	235.70	
0	230.49	236.48 ± 6.42
	243.25	
	209.59	
0.10	208.49	215.02 ± 10.39
	227.01	
	194.10	
0.25	189.55	195.99 ± 7.57
	204.33	
	140.34	
0.50	138.82	141.51 ± 3.43
	140.34	
	106.68	
1.0	115.22	112.75 ± 5.29
	116.35	
	97.51	
1.5	89.76	92.88 ± 4.09
	91.37	
	45.18	
2.5	47.29	45.87 ± 1.23
	45.15	
	20.54	
4.0	15.14	15.81 ± 4.43
	11.76	
	3.79	
5.0	6.75	4.72 ± 1.76
	3.61	

It is important to emphasize that the light conditions inside the Photo light Box, along with picture traits such as brightness, sharpness, and gamma levels, were adjusted to promote the overall maximum clearness of the solutions. These parameters were held constant for the entire experimentation with all particle sizes to maintain consistency. To appropriately handle null grayscale values, a power function (Eq. 1) was selected among several other types of functions to fit the data presented in Table S2. The fitting process was carried out using OriginPro 2023b software.

$$y = ab^x \qquad \text{(S1)}$$

which can be made explicit in y through:

$$x = \log c\left(\frac{y}{a}\right) \qquad \text{(S2)}$$

The resulting fit performed for the 20 nm particles is presented in Figure S1 along with estimated parameters and fitting quality. The optimization algorithm used to estimate the parameters was Levenberg-Marquadt.

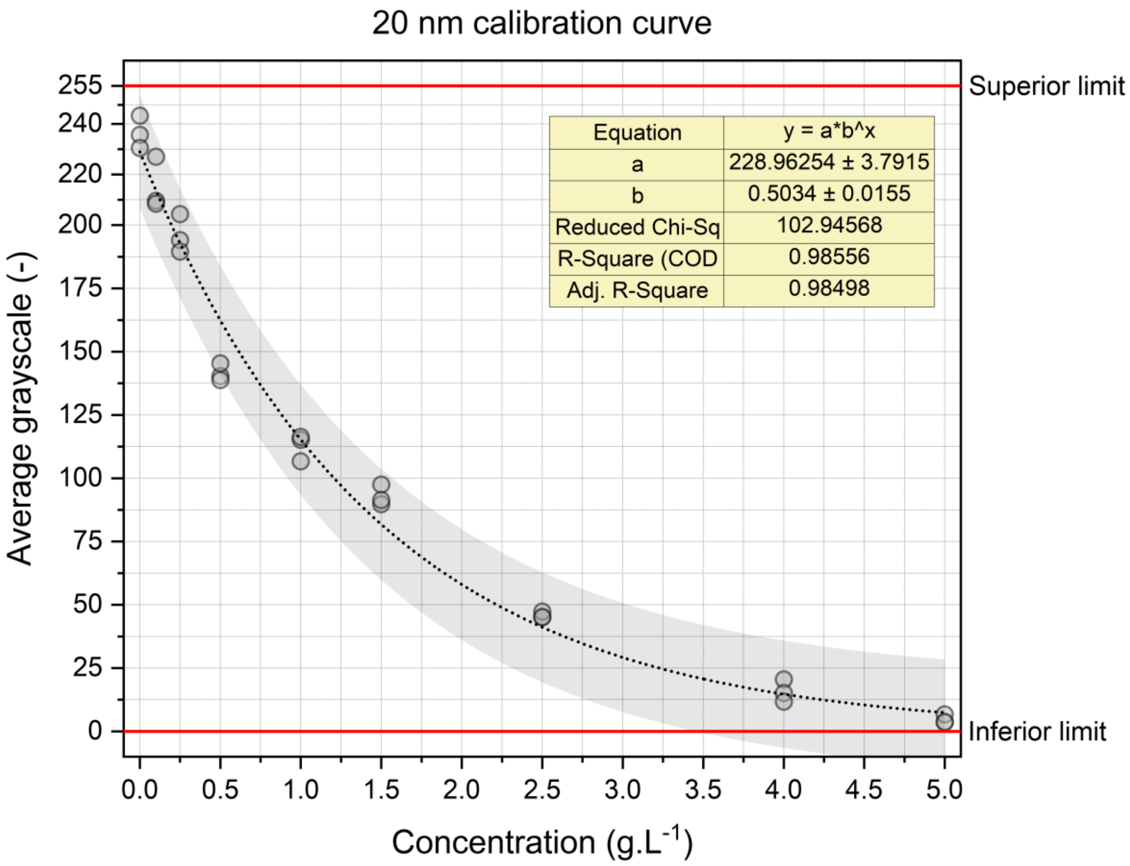

Figure S1. 20 nm SPIONs calibration curve

2 Calibration of 30 nm SPIONs

This section presents the mass concentration values of 30 nm SPIONs in aqueous solutions, along with their corresponding grayscale values. The measurements were conducted in triplicates and the arithmetic average was calculated for the entire length of the glass channel. Therefore, each row of the second columns of Table S3 contains the arithmetic average of grayscale intensity values for the entire length of the center of the channel.

Table S3. Experimental data for 30 nm SPIONs calibration

Mass concentration (g·L^{-1})	$\overline{G}$	$\overline{\overline{G}} \pm s$
	235.70	
0	230.49	236.48 ± 6.42
	243.25	
	218.59	
0.10	214.98	220.29 ± 6.33
	227.30	
	159.88	
0.25	168.45	167.33 ± 6.96
	173.67	
	109.13	
0.50	113.51	112.11 ± 2.58
	113.68	
	88.65	
1.0	101.46	94.74 ± 6.43
	94.10	
	41.24	
1.5	34.79	36.21 ± 4.49
	32.60	
	3.09	
2.5	2.50	2.24 ± 1.01
	1.13	
	2.14	
4.0	1.46	1.55 ± 0.55
	1.06	
	1.22	
5.0	1.37	1.20 ± 0.19
	1.00	

The same model was used to fit the experimental data in Table S3. The fitting process was carried out using OriginPro 2023b software. The resulting fit performed for the 30 nm particles is presented in Figure S2 along with estimated parameters and fitting quality. The optimization algorithm used to estimate the parameters was Levenberg-Marquadt.

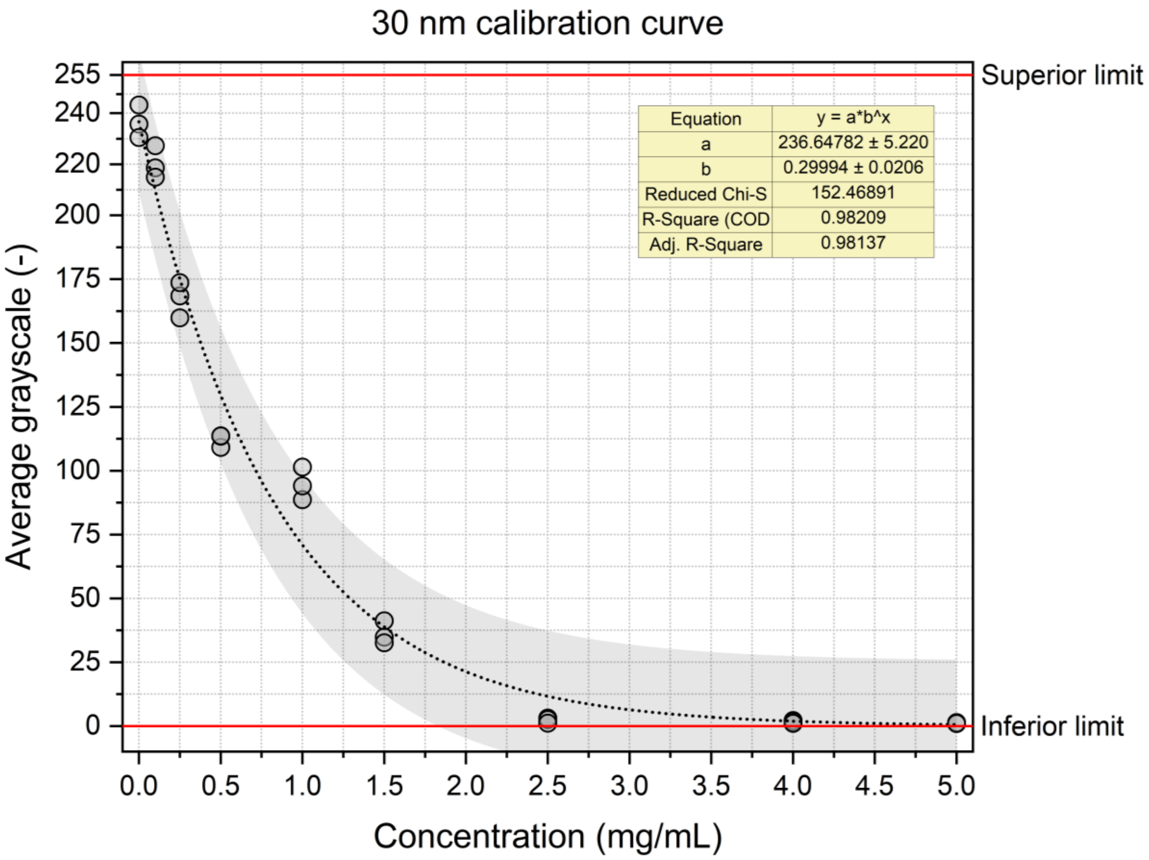

Figure S2. 30 nm SPIONs calibration curve

3 Experimental setup

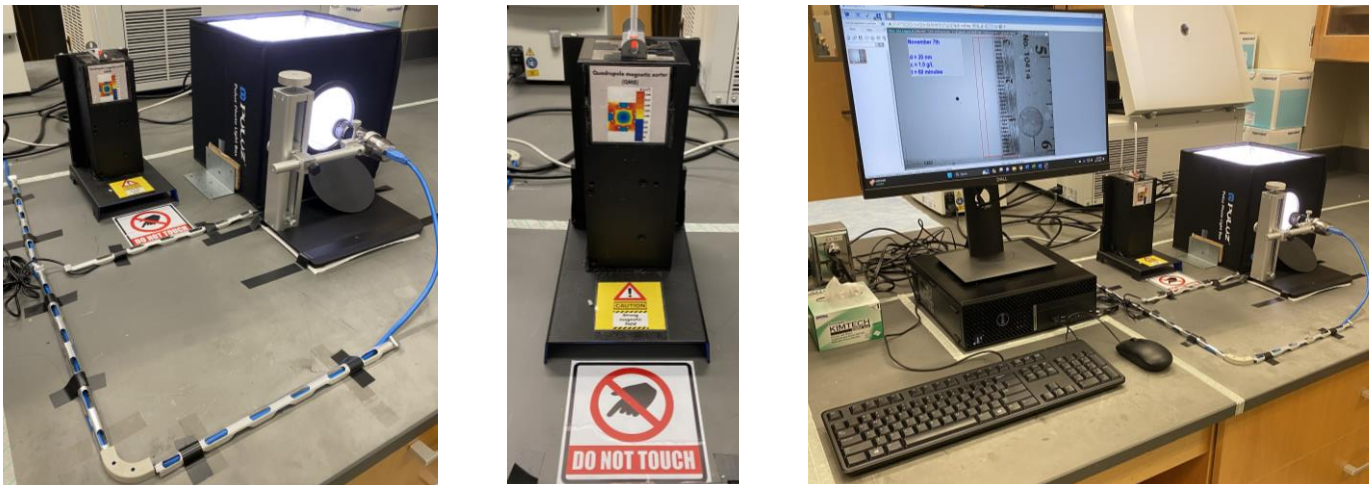

(a) (b) (c)

Figure S3. Experimental setup for magnetic separation of SPIONs using a QMS: (a) QMS and photo box, (b) frontal view of the QMS with a sample inserted in its bore, and (c) complete setup with image acquiring unit.

4 Magnetic separation control

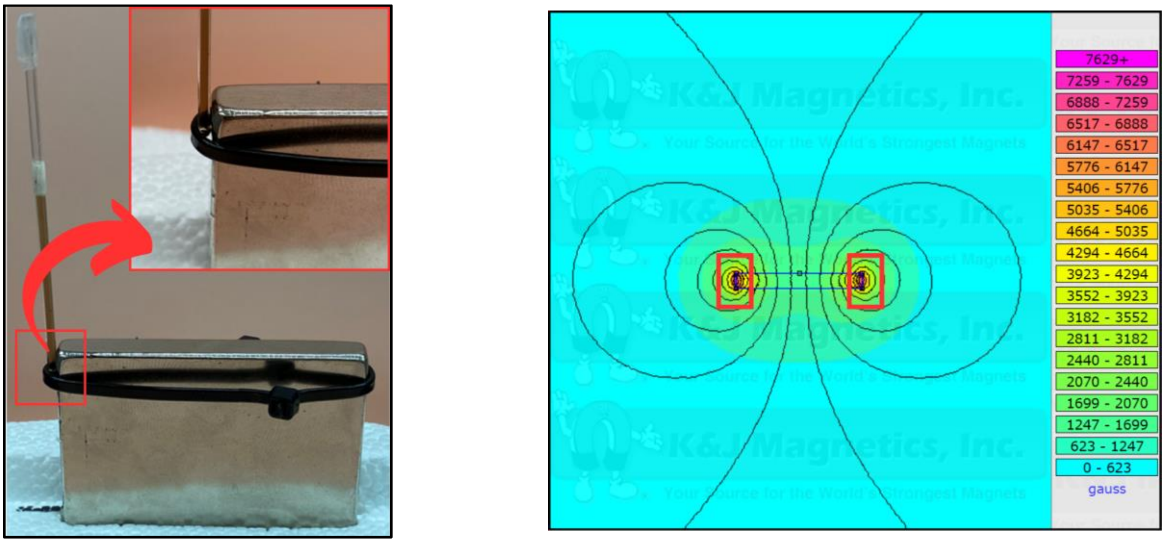

(a) (b)

Figure S4. The assembled magnetic separation control setup: (a) detail of the bottom of the sample placed as close as possible to the upper left corner of the NdFeB N42 magnet, where the magnetic field is most intense as highlighted in the field diagram (b) obtained from the magnet manufacturer's website.

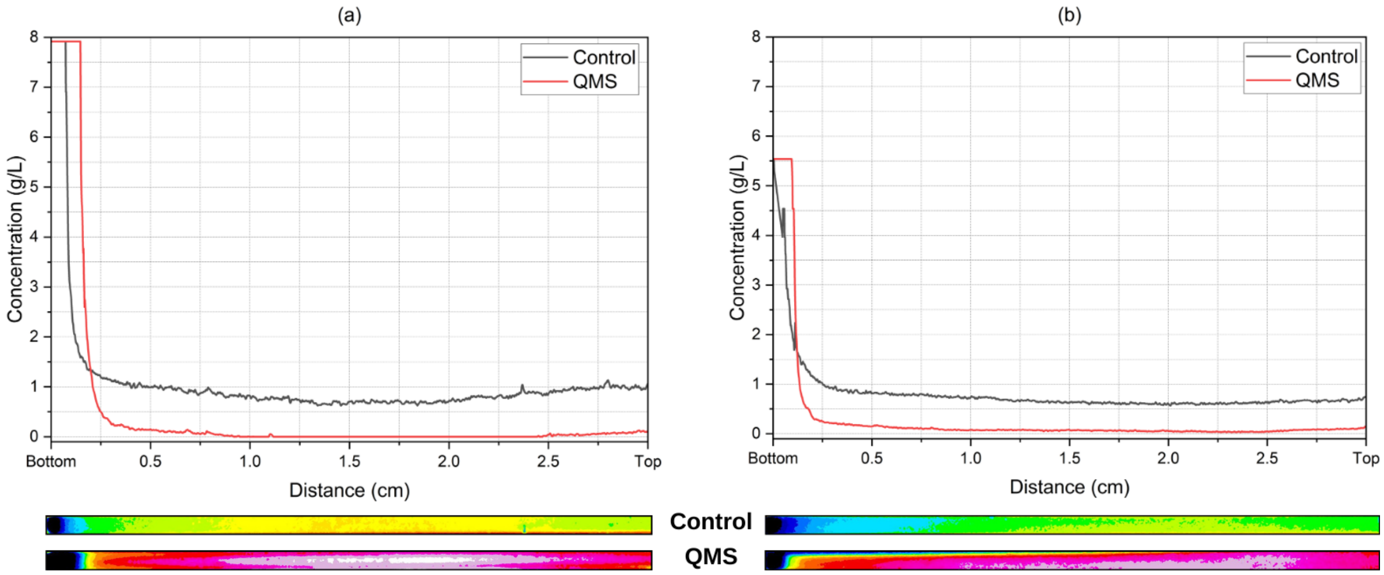

Figure S5: Magnetic separation of (a) 20 nm SPIONs and (b) 30 nm SPIONs at a initial concentration of 1.0 g·L^{-1} for 60 minutes using the control setup and the QMS. The black solid curve represents the concentration profile at the axial center of the sample using the apparatus shown in Figure S4, while the red solid curve represents the same quantity obtained using the QMS. Below the graphs one can find the 16-color concentration spectrum of each sample from left (sample bottom) to right (sample top) that undergone magnetic separation. The scales are the same ones as in Figures 4 and 5 for this figure's (a) and (b) parts, respectively.
